# Bis({1-[(1-imino­eth­yl)imino]­eth­yl}aza­nido-κ^2^ *N* ^1^,*N* ^5^)nickel(II) methanol monosolvate

**DOI:** 10.1107/S1600536812046958

**Published:** 2012-12-05

**Authors:** Yong-Qiang Xie, Jun-Jian Li, Ying Guo, You-Ming Zhang, Tai-Bao Wei

**Affiliations:** a Key Laboratory of Eco-Environment-Related Polymer Materials, Ministry of Education of China, Key Laboratory of Polymer Materials of Gansu Province, College of Chemistry and Chemical Engineering, Northwest Normal University, Lanzhou 730070, People’s Republic of China

## Abstract

The title compound, [Ni(C_4_H_8_N_3_)_2_]·CH_3_OH, contains two independent Ni^II^ atoms, each located on an inversion center and coordinated by four N atoms from two 1-[(1-imino­eth­yl)imino]­eth­yl}aza­nide ligands in a square-planar geometry. N—H⋯N, N—H⋯O and O—H⋯N hydrogen bonds link the complex mol­ecules and methanol solvent mol­ecules into a corrugated layer parallel to (001).

## Related literature

For structures and applications of related compounds, see: Aromi *et al.* (2011 ▶); Guzei *et al.* (2006 ▶); Kopylovich *et al.* (2007 ▶); Kryatov *et al.* (2001 ▶); Norrestam *et al.* (1983 ▶).
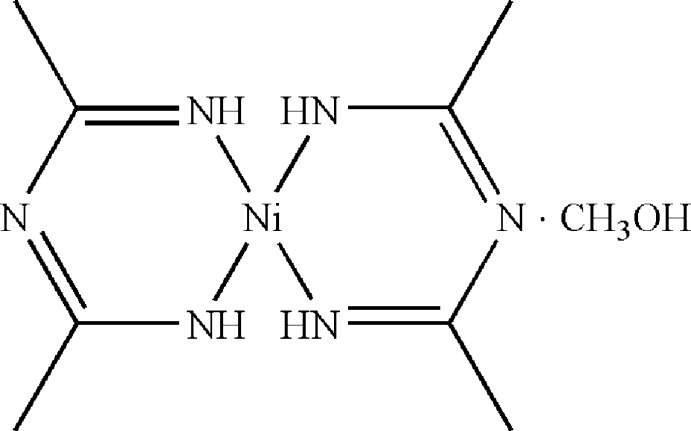


## Experimental

### 

#### Crystal data

[Ni(C_4_H_8_N_3_)_2_]·CH_4_O
*M* *_r_* = 287.02
Monoclinic, 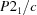

*a* = 9.2768 (7) Å
*b* = 11.4347 (3) Å
*c* = 12.9774 (3) Å
β = 92.961 (3)°
*V* = 1374.77 (11) Å^3^
*Z* = 4
Mo *K*α radiation
μ = 1.41 mm^−1^
*T* = 298 K
0.23 × 0.21 × 0.19 mm

#### Data collection

Bruker APEXII CCD diffractometer
Absorption correction: multi-scan (*SADABS*; Bruker, 2001 ▶) *T*_min_ = 0.603, *T*_max_ = 0.766
9293 measured reflections
2421 independent reflections
1738 reflections with *I* > 2σ(*I*)
*R*_int_ = 0.032

#### Refinement

*R*[*F* ^2^ > 2σ(*F* ^2^)] = 0.029
*wR*(*F* ^2^) = 0.082
*S* = 1.04
2421 reflections
163 parameters
H-atom parameters constrained
Δρ_max_ = 0.33 e Å^−3^
Δρ_min_ = −0.20 e Å^−3^

### 

Data collection: *APEX2* (Bruker, 2007 ▶); cell refinement: *SAINT* (Bruker, 2007 ▶); data reduction: *SAINT*; program(s) used to solve structure: *SHELXS97* (Sheldrick, 2008 ▶); program(s) used to refine structure: *SHELXL97* (Sheldrick, 2008 ▶); molecular graphics: *XP* in *SHELXTL* (Sheldrick, 2008 ▶); software used to prepare material for publication: *SHELXTL*.

## Supplementary Material

Crystal structure: contains datablock(s) I, global. DOI: 10.1107/S1600536812046958/hy2604sup1.cif

Structure factors: contains datablock(s) I. DOI: 10.1107/S1600536812046958/hy2604Isup2.hkl

Additional supplementary materials:  crystallographic information; 3D view; checkCIF report

## Figures and Tables

**Table 1:** Hydrogen-bond geometry (Å, °)

*D*—H⋯*A*	*D*—H	H⋯*A*	*D*⋯*A*	*D*—H⋯*A*
N1—H1⋯O1*A* ^i^	0.86	2.19	3.049 (3)	172
N2—H2⋯O1*A* ^ii^	0.86	2.23	3.079 (3)	169
N4—H4⋯N3^iii^	0.86	2.44	3.264 (3)	160
N5—H5⋯N3	0.86	2.31	3.153 (3)	165
O1*A*—H1*A*4⋯N6	0.82	1.90	2.711 (3)	172

Symmetry codes: (i) 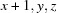; (ii) 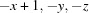; (iii) 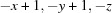.

**Fig. 1.**
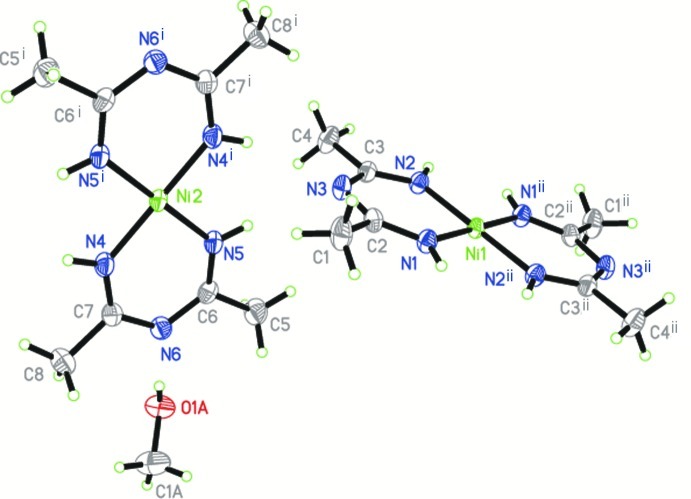
The molecular structure of the title complex. Displacement ellipsoids are drawn at the 30% probability level. [Symmetry codes: (i) 1-x, 1-y, -z; (ii) 2-x, -y, -z.]

**Fig. 2.**
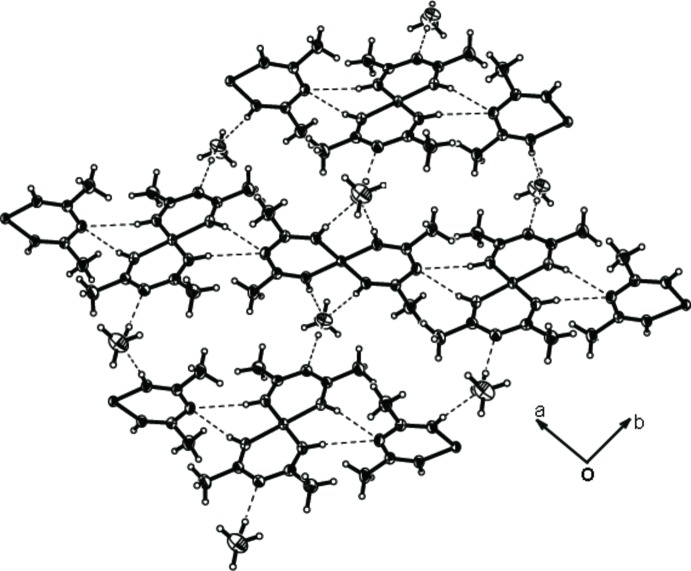
The two-dimensional supramolecular structure of the title complex.

## supplementary crystallographic information

### Comment

Acetonitrile is one of the common solvents that is widely used to study processes in solution. With most 3d-transition metal ions, acetonitrile behaves as a relatively weak monodentate ligand (Kopylovich *et al.*, 2007; Kryatov *et al.*, 2001), providing inorganic chemists with a perfect media for numerous reactions. As a whole, metal-promoted reactions of nitriles have proven to be a significant tool for the synthesis of diverse compounds, and several reviews on this topic have appeared in literatures in the past decades (Aromi *et al.*, 2011). However, a few reports showed the application of solvothermal synthetic techniques to reactions of nitriles with transition metal sources as a mean for the preparation of coordination compounds with molecular or extended structures (Guzei *et al.*, 2006). Here we study reactions of 3d-transition metal ions with acetonitrile in order to understand the reaction system and elucidate structural features of the resultant mononuclear metal complexes.

The asymmetric unit of the title compound contains two independent Ni^II^ atoms, each of which lies on an inversion center, and a methanol molecule, as shown in Fig. 1. Each Ni^II^ atom is in a square-planar geometry, coordinated by four N atoms from two 1-[(1-iminoethyl)imino]ethyl}azanide ligands. Two six-membered rings around the Ni^II^ atom is slightly distorted toward a boat conformation. In one six-membered ring, Ni1 and N2 atoms exist in the apex positions, while in the other ring Ni2 and N5 atoms do. The bond distances in the ligands are very similar to those observed for the simple acetamidine molecule (Norrestam *et al.*, 1983). In the crystal, the complex molecules are linked into a one-dimensional supramolecular architecture *via* N4—H4···N3^i^, N5—H5···N3 hydrogen bonds (Table 1) [symmetry code: (i) -*x*+1, -*y*+1, -*z*]. The one-dimensional architectures are further linked into a two-dimensional supramolecular structure with highly corrugated architecture *via* O—H···N and N—H···O hydrogen bonds between the ligands and the lattice methanol molecules, as shown in Fig. 2.

### Experimental

A mixture of Ni(NO_3_)_2_.6H_2_O (0.029 g, 0.1 mmol) in 12 ml of acetonitrile/methanol (3:1, *v*/*v*) and 0.1 ml of 2*M* NaOH solution was sealed in a Teflon-lined autoclave and heated under autogenous pressure to 160°C for 3 days and then allowed to cool to room temperature at a rate of 1°C per minute. Block-shaped tan crystals of the title complex were collected in 71% yield.

### Refinement

H atoms were positioned geometrically and refined as riding atoms, with C—H = 0.96, N—H = 0.86 and O—H = 0.82 Å and with *U*_iso_(H) = 1.2*U*_eq_(N) or 1.5*U*_eq_(C, O).

### Crystal data

**Table:**

[Ni(C_4_H_8_N_3_)_2_]·CH_4_O	*F*(000) = 608
*M**_r_* = 287.02	*D*_x_ = 1.387 Mg m^−^^3^*D*_m_ = 1.37 Mg m^−^^3^*D*_m_ measured by not measured
Monoclinic, *P*2_1_/*c*	Mo *K*α radiation, λ = 0.71073 Å
Hall symbol: -P 2ybc	Cell parameters from 9999 reflections
*a* = 9.2768 (7) Å	θ = 2.4–27.7°
*b* = 11.4347 (3) Å	µ = 1.41 mm^−^^1^
*c* = 12.9774 (3) Å	*T* = 298 K
β = 92.961 (3)°	Block, green
*V* = 1374.77 (11) Å^3^	0.23 × 0.21 × 0.19 mm
*Z* = 4	

### Data collection

**Table:**

Bruker APEXII CCD diffractometer	2421 independent reflections
Radiation source: fine-focus sealed tube	1738 reflections with *I* > 2σ(*I*)
Graphite monochromator	*R*_int_ = 0.032
φ and ω scans	θ_max_ = 25.0°, θ_min_ = 2.2°
Absorption correction: multi-scan (*SADABS*; Bruker, 2001)	*h* = −9→11
*T*_min_ = 0.603, *T*_max_ = 0.766	*k* = −13→13
9293 measured reflections	*l* = −15→15

### Refinement

**Table:**

Refinement on *F*^2^	Secondary atom site location: difference Fourier map
Least-squares matrix: full	Hydrogen site location: inferred from neighbouring sites
*R*[*F*^2^ > 2σ(*F*^2^)] = 0.029	H-atom parameters constrained
*wR*(*F*^2^) = 0.082	*w* = 1/[σ^2^(*F*_o_^2^) + (0.0344*P*)^2^ + 0.7499*P*] where *P* = (*F*_o_^2^ + 2*F*_c_^2^)/3
*S* = 1.04	(Δ/σ)_max_ < 0.001
2421 reflections	Δρ_max_ = 0.33 e Å^−^^3^
163 parameters	Δρ_min_ = −0.20 e Å^−^^3^
0 restraints	Extinction correction: *SHELXL97* (Sheldrick, 2008), Fc^*^=kFc[1+0.001xFc^2^λ^3^/sin(2θ)]^-1/4^
Primary atom site location: structure-invariant direct methods	Extinction coefficient: 0.051 (2)

### Special details

**Table:**

Geometry. All e.s.d.'s (except the e.s.d. in the dihedral angle between two l.s. planes) are estimated using the full covariance matrix. The cell e.s.d.'s are taken into account individually in the estimation of e.s.d.'s in distances, angles and torsion angles; correlations between e.s.d.'s in cell parameters are only used when they are defined by crystal symmetry. An approximate (isotropic) treatment of cell e.s.d.'s is used for estimating e.s.d.'s involving l.s. planes.
Refinement. Refinement of *F*^2^ against ALL reflections. The weighted *R*-factor *wR* and goodness of fit *S* are based on *F*^2^, conventional *R*-factors *R* are based on *F*, with *F* set to zero for negative *F*^2^. The threshold expression of *F*^2^ > σ(*F*^2^) is used only for calculating *R*-factors(gt) *etc*. and is not relevant to the choice of reflections for refinement. *R*-factors based on *F*^2^ are statistically about twice as large as those based on *F*, and *R*- factors based on ALL data will be even larger.

### Fractional atomic coordinates and isotropic or equivalent isotropic displacement parameters (Å^2^)

**Table:**

	*x*	*y*	*z*	*U*_iso_*/*U*_eq_	
C1	0.8993 (3)	0.3445 (2)	0.0859 (3)	0.0599 (8)	
H1A	0.9269	0.3305	0.1572	0.090*	
H1B	0.8074	0.3832	0.0810	0.090*	
H1C	0.9704	0.3929	0.0558	0.090*	
C2	0.8886 (3)	0.2297 (2)	0.02917 (19)	0.0394 (6)	
C3	0.7796 (3)	0.1309 (2)	−0.10996 (19)	0.0383 (6)	
C4	0.6614 (3)	0.1373 (3)	−0.1938 (2)	0.0578 (8)	
H4A	0.6647	0.0688	−0.2365	0.087*	
H4B	0.6747	0.2057	−0.2351	0.087*	
H4C	0.5695	0.1413	−0.1632	0.087*	
N1	0.9732 (2)	0.14471 (17)	0.05897 (16)	0.0391 (5)	
H1	1.0248	0.1577	0.1148	0.047*	
N2	0.8599 (2)	0.03823 (17)	−0.10115 (15)	0.0379 (5)	
H2	0.8460	−0.0129	−0.1493	0.045*	
N3	0.7895 (2)	0.22670 (18)	−0.05010 (16)	0.0423 (5)	
Ni1	1.0000	0.0000	0.0000	0.03187 (16)	
C5	0.4642 (3)	0.1559 (2)	0.1102 (3)	0.0606 (8)	
H5A	0.3862	0.1150	0.0746	0.091*	
H5B	0.4685	0.1344	0.1818	0.091*	
H5C	0.5535	0.1357	0.0805	0.091*	
C6	0.4397 (3)	0.2853 (2)	0.1004 (2)	0.0407 (6)	
C7	0.3098 (3)	0.4450 (2)	0.1593 (2)	0.0402 (6)	
C8	0.2071 (4)	0.4860 (2)	0.2380 (2)	0.0561 (8)	
H8A	0.2570	0.4901	0.3046	0.084*	
H8B	0.1282	0.4319	0.2407	0.084*	
H8C	0.1707	0.5620	0.2190	0.084*	
N4	0.3630 (2)	0.52029 (17)	0.09697 (17)	0.0423 (6)	
H4	0.3296	0.5902	0.1014	0.051*	
N5	0.5126 (2)	0.34436 (18)	0.03568 (17)	0.0406 (5)	
H5	0.5765	0.3050	0.0047	0.049*	
N6	0.3410 (2)	0.32978 (18)	0.16225 (16)	0.0431 (5)	
Ni2	0.5000	0.5000	0.0000	0.03481 (17)	
C1A	0.1695 (5)	0.1778 (3)	0.3585 (3)	0.0868 (12)	
H1A1	0.2617	0.1556	0.3894	0.130*	
H1A2	0.0962	0.1268	0.3826	0.130*	
H1A3	0.1484	0.2570	0.3771	0.130*	
O1A	0.1728 (2)	0.16916 (17)	0.25194 (14)	0.0601 (6)	
H1A4	0.2242	0.2210	0.2302	0.090*	

### Atomic displacement parameters (Å^2^)

**Table:**

	*U*^11^	*U*^22^	*U*^33^	*U*^12^	*U*^13^	*U*^23^
C1	0.0588 (19)	0.0430 (17)	0.076 (2)	0.0143 (15)	−0.0105 (16)	−0.0205 (15)
C2	0.0371 (15)	0.0335 (14)	0.0476 (15)	0.0039 (12)	0.0022 (12)	−0.0055 (11)
C3	0.0365 (14)	0.0359 (14)	0.0422 (14)	0.0038 (12)	−0.0001 (11)	0.0030 (11)
C4	0.0597 (19)	0.0530 (18)	0.0582 (18)	0.0117 (15)	−0.0199 (15)	−0.0022 (14)
N1	0.0408 (12)	0.0349 (11)	0.0408 (12)	0.0056 (10)	−0.0048 (10)	−0.0067 (9)
N2	0.0414 (13)	0.0322 (11)	0.0395 (12)	0.0048 (10)	−0.0037 (9)	−0.0048 (9)
N3	0.0400 (13)	0.0359 (12)	0.0502 (13)	0.0094 (10)	−0.0044 (10)	−0.0036 (10)
Ni1	0.0320 (3)	0.0273 (3)	0.0360 (3)	0.00400 (18)	−0.00116 (18)	−0.00203 (18)
C5	0.064 (2)	0.0380 (16)	0.081 (2)	0.0033 (15)	0.0155 (17)	0.0081 (15)
C6	0.0399 (15)	0.0325 (13)	0.0490 (16)	−0.0001 (12)	−0.0043 (12)	0.0039 (12)
C7	0.0347 (15)	0.0402 (15)	0.0454 (15)	−0.0010 (12)	−0.0012 (12)	−0.0033 (12)
C8	0.0558 (18)	0.0507 (19)	0.063 (2)	−0.0025 (14)	0.0164 (15)	−0.0092 (14)
N4	0.0429 (13)	0.0323 (12)	0.0521 (13)	0.0071 (10)	0.0051 (11)	−0.0004 (10)
N5	0.0389 (13)	0.0343 (12)	0.0484 (12)	0.0081 (10)	0.0023 (10)	0.0000 (10)
N6	0.0426 (13)	0.0376 (12)	0.0493 (13)	−0.0005 (10)	0.0061 (11)	0.0016 (10)
Ni2	0.0339 (3)	0.0292 (3)	0.0413 (3)	0.00597 (19)	0.00096 (19)	0.00100 (19)
C1A	0.124 (4)	0.078 (3)	0.058 (2)	−0.018 (2)	0.006 (2)	0.0053 (19)
O1A	0.0711 (15)	0.0590 (13)	0.0505 (12)	−0.0232 (11)	0.0049 (10)	−0.0076 (10)

### Geometric parameters (Å, º)

**Table:**

C1—C2	1.506 (3)	C5—H5C	0.9600
C1—H1A	0.9600	C6—N5	1.296 (3)
C1—H1B	0.9600	C6—N6	1.348 (3)
C1—H1C	0.9600	C7—N4	1.297 (3)
C2—N1	1.295 (3)	C7—N6	1.349 (3)
C2—N3	1.344 (3)	C7—C8	1.507 (4)
C3—N2	1.296 (3)	C8—H8A	0.9600
C3—N3	1.344 (3)	C8—H8B	0.9600
C3—C4	1.506 (3)	C8—H8C	0.9600
C4—H4A	0.9600	N4—Ni2	1.848 (2)
C4—H4B	0.9600	N4—H4	0.8600
C4—H4C	0.9600	N5—Ni2	1.841 (2)
N1—Ni1	1.8452 (19)	N5—H5	0.8600
N1—H1	0.8600	Ni2—N5^ii^	1.841 (2)
N2—Ni1	1.851 (2)	Ni2—N4^ii^	1.848 (2)
N2—H2	0.8600	C1A—O1A	1.388 (4)
Ni1—N1^i^	1.8452 (19)	C1A—H1A1	0.9600
Ni1—N2^i^	1.851 (2)	C1A—H1A2	0.9600
C5—C6	1.501 (3)	C1A—H1A3	0.9600
C5—H5A	0.9600	O1A—H1A4	0.8200
C5—H5B	0.9600		
			
C2—C1—H1A	109.5	H5A—C5—H5C	109.5
C2—C1—H1B	109.5	H5B—C5—H5C	109.5
H1A—C1—H1B	109.5	N5—C6—N6	125.6 (2)
C2—C1—H1C	109.5	N5—C6—C5	119.2 (3)
H1A—C1—H1C	109.5	N6—C6—C5	115.2 (2)
H1B—C1—H1C	109.5	N4—C7—N6	125.3 (3)
N1—C2—N3	126.1 (2)	N4—C7—C8	119.4 (2)
N1—C2—C1	119.0 (2)	N6—C7—C8	115.3 (2)
N3—C2—C1	114.9 (2)	C7—C8—H8A	109.5
N2—C3—N3	126.4 (2)	C7—C8—H8B	109.5
N2—C3—C4	119.8 (2)	H8A—C8—H8B	109.5
N3—C3—C4	113.8 (2)	C7—C8—H8C	109.5
C3—C4—H4A	109.5	H8A—C8—H8C	109.5
C3—C4—H4B	109.5	H8B—C8—H8C	109.5
H4A—C4—H4B	109.5	C7—N4—Ni2	129.69 (19)
C3—C4—H4C	109.5	C7—N4—H4	115.2
H4A—C4—H4C	109.5	Ni2—N4—H4	115.2
H4B—C4—H4C	109.5	C6—N5—Ni2	129.70 (18)
C2—N1—Ni1	129.87 (18)	C6—N5—H5	115.1
C2—N1—H1	115.1	Ni2—N5—H5	115.1
Ni1—N1—H1	115.1	C6—N6—C7	120.2 (2)
C3—N2—Ni1	129.38 (18)	N5^ii^—Ni2—N5	180.00 (13)
C3—N2—H2	115.3	N5^ii^—Ni2—N4	90.74 (9)
Ni1—N2—H2	115.3	N5—Ni2—N4	89.26 (9)
C3—N3—C2	119.1 (2)	N5^ii^—Ni2—N4^ii^	89.26 (9)
N1—Ni1—N1^i^	180.00 (13)	N5—Ni2—N4^ii^	90.74 (9)
N1—Ni1—N2	88.73 (9)	N4—Ni2—N4^ii^	180.0
N1^i^—Ni1—N2	91.27 (9)	O1A—C1A—H1A1	109.5
N1—Ni1—N2^i^	91.27 (9)	O1A—C1A—H1A2	109.5
N1^i^—Ni1—N2^i^	88.73 (9)	H1A1—C1A—H1A2	109.5
N2—Ni1—N2^i^	180.00 (17)	O1A—C1A—H1A3	109.5
C6—C5—H5A	109.5	H1A1—C1A—H1A3	109.5
C6—C5—H5B	109.5	H1A2—C1A—H1A3	109.5
H5A—C5—H5B	109.5	C1A—O1A—H1A4	109.5
C6—C5—H5C	109.5		
			
N3—C2—N1—Ni1	6.0 (4)	N6—C7—N4—Ni2	−3.4 (4)
C1—C2—N1—Ni1	−173.5 (2)	C8—C7—N4—Ni2	174.9 (2)
N3—C3—N2—Ni1	6.1 (4)	N6—C6—N5—Ni2	−4.4 (4)
C4—C3—N2—Ni1	−173.7 (2)	C5—C6—N5—Ni2	175.8 (2)
N2—C3—N3—C2	−1.7 (4)	N5—C6—N6—C7	0.6 (4)
C4—C3—N3—C2	178.2 (2)	C5—C6—N6—C7	−179.6 (2)
N1—C2—N3—C3	−4.4 (4)	N4—C7—N6—C6	3.3 (4)
C1—C2—N3—C3	175.1 (2)	C8—C7—N6—C6	−175.1 (2)
C2—N1—Ni1—N2	−1.8 (2)	C6—N5—Ni2—N4	3.5 (2)
C2—N1—Ni1—N2^i^	178.2 (2)	C6—N5—Ni2—N4^ii^	−176.5 (2)
C3—N2—Ni1—N1	−3.9 (2)	C7—N4—Ni2—N5^ii^	−179.8 (2)
C3—N2—Ni1—N1^i^	176.1 (2)	C7—N4—Ni2—N5	0.2 (2)

Symmetry codes: (i) −*x*+2, −*y*, −*z*; (ii) −*x*+1, −*y*+1, −*z*.

### Hydrogen-bond geometry (Å, º)

**Table:**

*D*—H···*A*	*D*—H	H···*A*	*D*···*A*	*D*—H···*A*
N1—H1···O1*A*^iii^	0.86	2.19	3.049 (3)	172
N2—H2···O1*A*^iv^	0.86	2.23	3.079 (3)	169
N4—H4···N3^ii^	0.86	2.44	3.264 (3)	160
N5—H5···N3	0.86	2.31	3.153 (3)	165
O1*A*—H1*A*4···N6	0.82	1.90	2.711 (3)	172

Symmetry codes: (ii) −*x*+1, −*y*+1, −*z*; (iii) *x*+1, *y*, *z*; (iv) −*x*+1, −*y*, −*z*.
